# The evolution of group size and kin structure in complex societies

**DOI:** 10.1098/rsos.260096

**Published:** 2026-10-28

**Authors:** Sheng-Feng Shen, Dustin R. Rubenstein, Ming Liu

**Affiliations:** ^1^ Biodiversity Research Center, Academia Sinica, Taipei City, Taiwan; ^2^ Department of Ecology, Evolution and Environmental Biology, Columbia University, New York, NY, USA; ^3^ Department of Biology, University of Oxford, Oxford, UK

**Keywords:** insider-outsider conflict, kin structure, group size, inclusive fitness, cooperative breeding

## Abstract

Molecular and pedigree data reveal substantial variation in genetic relatedness within social groups across animals. Yet kin selection theory typically treats relatedness as a fixed parameter rather than an emergent property of group dynamics. We develop a demographically explicit model in which group size fluctuates through births, dispersal, mortality and floater immigration. Residents and potential joiners resolve conflict over membership, with joining probabilities set by inclusive-fitness effects at demographic equilibrium. Membership control jointly shapes group size distributions, stability and relatedness. Under insider control, unrelated outsiders are admitted primarily to small, low-productivity groups, so mean relatedness rises with group size and stays high in larger groups. Groups are most stable near the *per capita* productivity optimum, with equilibrium sizes slightly above it. Low fecundity relative to resident mortality increases floater reliance, promoting mixed-kin groups and reducing relatedness. Under outsider control, groups grow larger by admitting unrelated floaters, and relatedness remains low. By treating kin structure as an emergent outcome of demographic processes and membership decisions rather than a fixed parameter, our framework links relatedness to the processes that form and regulate social groups, offering new insight into the evolution of sociality.

## 1. Introduction

Since the concept of inclusive fitness was proposed more than half a century ago, the genetic or kin structure of social groups has long been a key predictor for the role that kin selection plays in the evolution and maintenance of animal societies [1–6]. Researchers have developed sophisticated mathematical frameworks to analyse how specific life history traits and demographic processes shape the genetic composition of populations and their spatial distribution, providing quantitative predictions about the conditions that favour cooperation [7–10]. However, while some of these frameworks do explicitly derive relatedness from demographic processes (e.g. [8]), almost all assume a simplistic group formation process of free entry such that groups contain a fixed number of breeding vacancies and whoever finds the vacancy first can occupy it.

Although it is well recognized that the costs and benefits of grouping vary with group size [11], past models have largely assumed that even if group members would suffer from the addition of other individuals, groups would still not adopt any strategies to influence the entry of outsiders. Thus, while previous models have greatly enhanced our understanding of the range of kin structures that can favour the evolution of helping behaviour, simplistic assumptions of fixed breeding vacancies and free entry have limited our ability to understand how demographic dynamics (e.g. group size regulation and membership stability) interact with kin selection to shape group formation processes. This gap is particularly problematic when considering highly social species where admission to groups is actively controlled, and group composition is dynamically maintained through complex social interactions [11–16].

An important but largely overlooked theoretical framework for understanding group formation is the theory of insider-outsider conflict [6,17–19]. Insider-outsider conflict describes the tension between current group members (insiders) and potential joiners (outsiders). Current group members often have aligned interests in maintaining the most productive group size. However, as long as joining a group is more beneficial than remaining solitary, an outsider will try to join a group, even if doing so lowers the group's productivity by making it too large. As a result, observed group sizes are often larger than the most productive size [6,20]. The concept of insider-outsider conflict has been used to predict the influence of relatedness on observed group size, finding that even when individuals prefer to form groups with kin, unrelated individuals can still force their way into a social group [17,18,21]. As a consequence, these models predict that related outsiders are more likely to be accepted if the group size is larger than the most productive size under insider-controlled group membership. However, these models also predict that related outsiders are more likely to refrain from joining a group if doing so reduces the direct fitness of their relatives in the group [22]. By contrast, unrelated individuals may attempt to join groups even if their addition would reduce the average productivity of the group, provided that the fitness benefits of group living exceed those of solitary reproduction. Such unrelated outsiders are likely to face resistance from insiders who would incur fitness costs from their inclusion, leading to potential repelling behaviours and conflicts over group membership.

Another key limitation of previous insider-outsider models is that they have exclusively focused on how kinship influences the resolution of group membership conflict when the group size is larger than the most productive size [17,18,21]. Yet, kin structure within groups fluctuates owing to a variety of demographic and life history factors, both of which influence the recruitment of offspring born in the group as well as the number of potential joining unrelated floaters. These factors are likely to impact kin structure before groups reach their most productive size. In other words, previous insider-outsider conflict models do not make predictions about genetic relatedness among group members when the group size is equal to or smaller than the most productive size. The development of demographically explicit models has shown that it is important to consider the feedbacks between individual strategies, including dispersal [23], helping and harming behaviour [8], as well as population dynamics [24].

These approaches expand upon the foundational inclusive-fitness machinery developed for structured populations [2,3,8,25]. Building on this machinery, many group and population properties, such as within-group relatedness and the pay-off of dispersing, should not be treated as fixed parameters, as has been assumed in previous models of insider-outsider conflict.

The increasing accumulation of molecular data in social species has shown that there is a great deal of variation in the genetic relatedness among group members both within and across different populations and species [26–32]. Nevertheless, whether individuals can employ behavioural strategies to influence the kin structure of social groups remains poorly explored in theoretical models. Instead, variation in within-group genetic relatedness is often treated as an explanatory parameter for individual strategies, such as the level of parental investment, social conflict or reproductive skew [33–36]. Similarly, the presence of mixed-kin groups comprising both kin and non-kin has often been interpreted as evidence supporting the role of direct fitness in promoting sociality [37–41]. However, such mixed-kin groups can also arise from demographic processes—for example, when low fecundity limits the availability of kin, or when high floater immigration dilutes within-group relatedness—which does not preclude a concurrent role for direct benefits. Yet, surprisingly few empirical studies have investigated the group formation process (but see [31,42]) or the causes of variation in group-size and kin-structure distributions observed in nature (but see [29,30]). This gap may be partly owing to the lack of an appropriate theoretical framework for addressing these issues. Here, we develop a novel demographically explicit model to explore how group membership control, grouping benefits and demographic factors jointly influence the kin structure and distribution of group sizes in a population of social organisms.

## 2. The model

We focus on an asexual population occupying a large number of discrete patches or territories, each of which contains a variable number of individuals making up the group (see figure 1 for model overview). Group size, *n*, can vary between 0 and n_max_, and is influenced by the demographic processes of reproduction, dispersal, mortality and immigration of floaters. To ensure that our model remains tractable while maintaining biological relevance, we must set some upper limit to group size. The value of n_max_ enters the model in two ways: it truncates the state space, and it appears in the baseline productivity function defined below, where it sets the group size at which productivity vanishes. All results reported here use *n*_max_ = 9; at this value, and given the density-dependent productivity described below, group size attains the upper limit with negligible probability at equilibrium. Generations are overlapping, and we model the demographics of our population in continuous time, such that events occur one-at-a-time, sequentially, as in a continuous-time, Moran-style event-based process; unlike the classical Moran model, however, our population size is not fixed [43].

**Figure 1.**
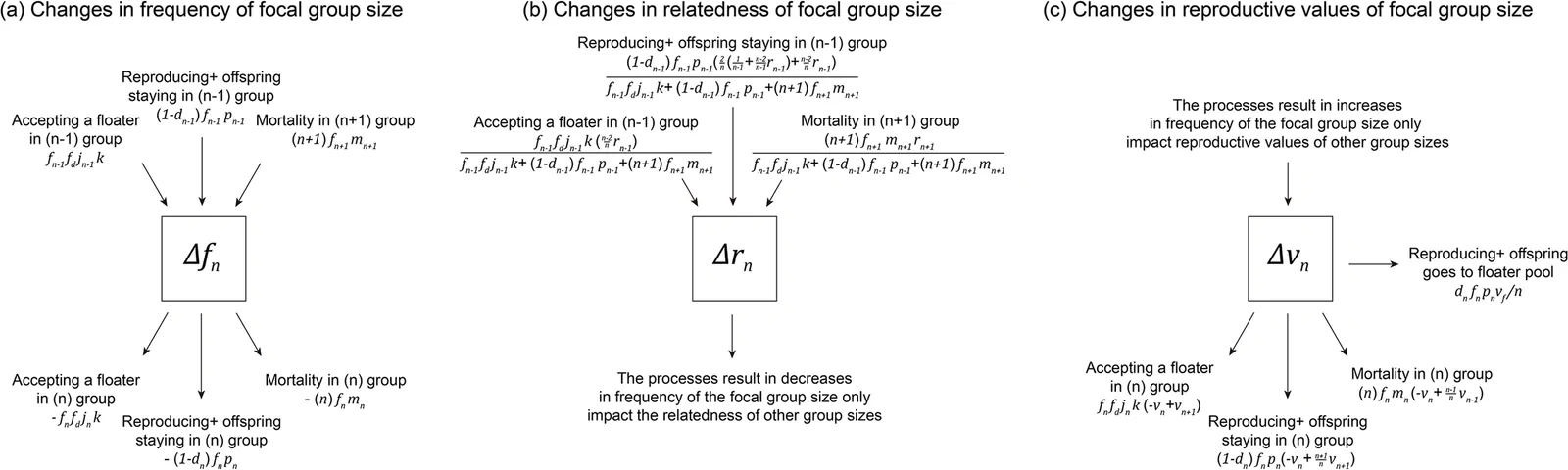
Schematic overview of the demographic bookkeeping used in the model. Panels show the events contributing to (a) changes in the frequency of groups of size *n* (i.e. Δ*f_n_*), (b) changes in mean within-group relatedness for groups of size *n* (i.e. Δ*r_n_*) and (c) changes in reproductive values for individuals in groups of size *n* (i.e. Δ*v_n_*). Arrows pointing towards the central box indicate events that create (or increase the weight of) the focal class, whereas arrows pointing away indicate events that remove (or decrease the weight of) the focal class. Events include production of a non-dispersing offspring (which increases group size by one), acceptance of an outsider (offspring or floater; which increases group size by one) and mortality (which decreases group size by one). The floater pool is treated as a separate class and is not depicted here (see the supplementary material for full equations).

Mortality parameters *m* (residents) and *m*_*f*  _(floaters) are *per capita* hazard rates (*time*^−1^), not discrete-time probabilities, and therefore are not bounded by 1. For simplicity, we assume a constant resident mortality hazard across group sizes and set *m*  =  1, so that time is measured in units of an individual resident's expected lifespan (i.e. 1/*m*). Floaters (i.e. individuals that are not part of any social group) experience mortality at hazard rate *m_f_*, which may differ from *m*. In a group of size *n*, death events occur at total rate *nm*, and each death event removes exactly one randomly chosen individual; consequently, group size changes by at most one individual per event (i.e. n→n−1). New individuals are produced at a rate *p*(*n*) in a group of size *n*, with all members of the group having equal expected fecundity (i.e. expected *per capita* fecundity is *p*(*n*)/*n*). To capture variation among populations or species in overall reproductive output, we write group productivity as *p*(*n*)  =  *g* *p*_0_(*n*), where *g* > 0 is a fecundity multiplier and *p*_0_(*n*) is a baseline productivity function. Throughout, we use *p*_0_(*n*) = 0.2 *n*^1.5^ (*n*_max_ − *n*)^0.75^ with *n*_max_ = 9, so that *per capita* productivity peaks at an intermediate group size and vanishes at *n* = *n*_max_; the fecundity multiplier is *g* = 1 unless stated otherwise. All baseline parameter values are listed in table S2 in the supplementary material.

We assume that *per capita* productivity *p*(*n*)/*n* initially increases but later decreases with group size (figure 2), and we refer to the group size that maximizes *per capita* productivity as the most productive group size. Because *g* rescales *p*(*n*) multiplicatively, varying *g* changes the magnitude of productivity but does not shift the location of this *per capita* productivity peak. This positive density-dependency pattern, often called an Allee effect, is commonly observed in social vertebrates and invertebrates [44–46]. Each newly produced individual may either join its natal group or disperse and enter a pool of floaters with a dispersal probability *d(n)*. We note that each newly produced offspring is itself a potential joiner, and so an outsider in this framework. Dispersed offspring enter a non-reproductive floater pool, sampling existing territories randomly until they find an empty territory or a group that they are willing to join. Therefore, floaters, on average, are unrelated to the members of the group they join. Thus, membership control cannot preferentially accept kin among foreign floaters; instead, it primarily mediates the relative contributions of natal recruitment (kin) versus floater immigration (non-kin) to group growth. A floater encounters groups of size *n* at a rate proportional to their frequency in the population, with a constant encounter rate k. The function *j(n)* specifies the probability of joining a foreign group of any given size *n* (including, for *n* = 0, the probability of occupying an empty territory). Floaters experience mortality at hazard rate *m_f_*. When presenting ‘joining rates’, we refer to event rates per unit time (such as *p*(*n*) [1 − *d*(*n*)] for natal recruitment and *f_d_kj*(*n*) for floater immigration) rather than to probabilities. Determining the joining probabilities *j*(*n*), together with the dispersal probabilities *d*(*n*), is the central output of our model.

**Figure 2.**
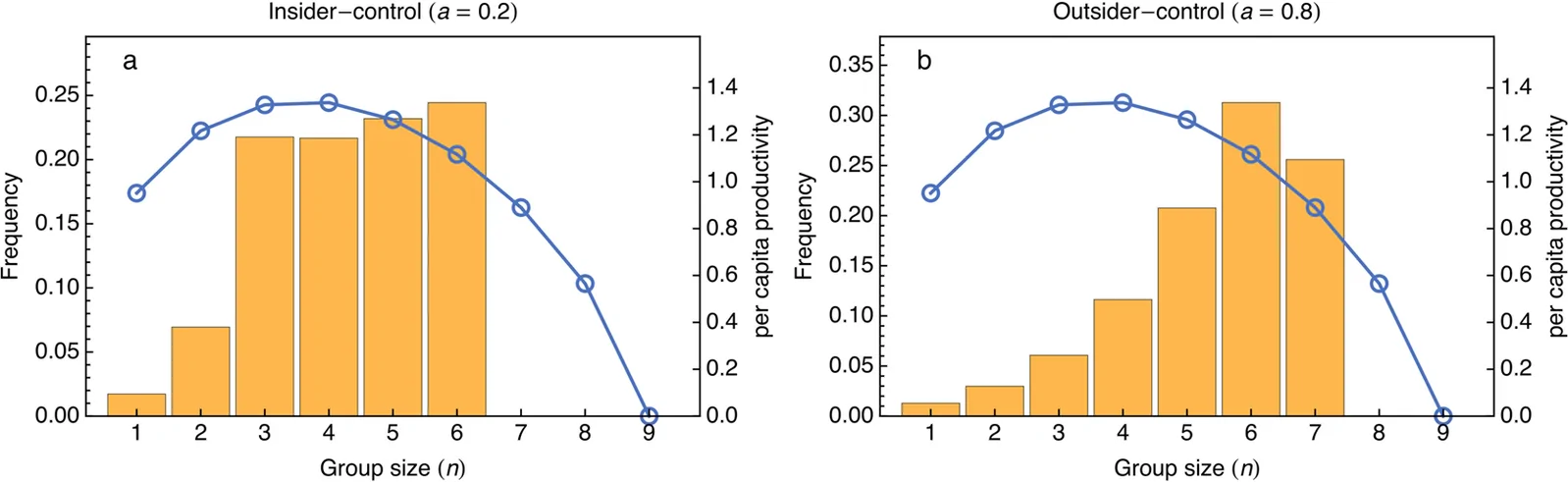
Example equilibrium distributions of group sizes and the assumed *per capita* productivity curve under two membership-control regimes. Bars show the equilibrium frequency *f*(*n*) of groups of size *n* (left y-axis). The line shows *per capita* productivity *p*(*n*)/*n* as a function of group size (right y-axis), which increases at small *n* and declines at large *n*. (a) Insider-control scenario (*a*  =  0.2). (b) Outsider-control scenario (*a*  =  0.8). All other parameters are held at the baseline values used throughout unless stated otherwise.

In light of the potential conflicts of interest over group membership between insiders and outsiders, we determine the self-consistent equilibrium joining probabilities for individuals joining both natal and foreign groups. We assume that at equilibrium, the probability of a newly produced juvenile or of a floater joining a group (of any given size) depends on the balance between the inclusive-fitness benefits of joining for the focal individual and the inclusive-fitness costs this outcome would impose on the existing members of the group. To be precise, when considering an individual joining a group of size *n*, we take a weighted sum of the inclusive-fitness consequences of joining for the focal (outsider) individual, plus *n* times the mean inclusive-fitness consequences for existing (insider) members, weighting outsider and insider terms by *a* and 1 − *a*, respectively. The probability of joining then depends on the value of this weighted sum. The parameter *a* determines the relative degree of outsider versus insider control over joining: a value of *a* = 0 implies that joining is determined entirely by the pay-offs to existing insiders, whereas a value of *a* = 1 implies that joining is determined entirely by the pay-offs to the outsider.

The simplest assumption would be that the focal individual (the outsider) joins with a probability of 1 if the sum is positive (i.e. if the inclusive-fitness benefits it stands to gain from group membership exceed the costs that will be suffered by the insiders by a ratio of at least (1 − *a*) : *a*), and with a probability of 0 if the sum is negative. Following McNamara *et al.* [47], we incorporate a degree of uncertainty in the outcome of the insider-outsider conflict. Specifically, letting Δ(*n*)  =  *a* Δ*I*_out_(*n*) + (1 − *a*) *n* Δ*I*_in_(*n*) denote the net inclusive-fitness effect of a joining event, we map Δ(*n*) to a joining probability using a logistic function:

$$P_{join}\;\left(\Delta \right)=\frac{1}{1+\exp\left\{-\frac{\Delta }{\epsilon }\right\}}\;.$$

Here *ε* > 0 controls uncertainty/steepness (smaller *ε* approaches a step function), and exp(⋅) is the exponential function with Euler's number as the base. This smooth mapping both captures decision errors and improves numerical stability when iterating towards a self-consistent equilibrium. We use *ε* = 0.025 throughout; the implementation evaluates the equivalent form (1 + tanh(Δ/err))/2 with err = 2*ε* = 0.05.

To derive equilibrium dispersal and joining probabilities, we adopt an iterative best-response procedure, treating evolutionary change as occurring on a time-scale distinct from that of the underlying demographic dynamics. The procedure repeats three steps until convergence.

We adopt a boundary convention at *n* = 0. An empty territory has no insiders to exert membership control, so the joining decision is determined entirely by the prospective floater's own pay-off. Throughout, *v*(*n*) denotes the reproductive value of an individual in a group of size *n* and *v_F_* that of a floater; both are obtained at the demographic equilibrium described below and are derived in the supplementary material. We therefore set *Δ*(0) = *ΔI*_out_(0) = *v*(1) − *v_F_*, equivalent to imposing *a* = 1 at *n* = 0. This convention avoids the otherwise indifferent rule *j*_BR_(0) = *P*_join_(0) = 1/2 that would result from naive substitution into the weighted decision rule at full insider control (*a* = 0), where the insider term vanishes for the trivial reason that there are no insiders. Given a candidate strategy profile {*d*(*t*)(*n*), *j*(*t*)(*n*)} at iteration *t*, we first solve numerically for the demographic equilibrium implied by these strategies, obtaining the stationary group-size distribution *f*(*n*), floater density *f*_d_, mean within-group relatedness *r*(*n*) and reproductive values *v*(n) and *v*_F_. At this demographic equilibrium, we next evaluate, for each group size *n*, the inclusive-fitness change Δ(*n*) associated with an outsider joining a group of size *n*, and substitute it into the logistic mapping introduced above to obtain the *best-response* joining probability *j*_BR_(*n*) = *P*_join_(Δ(*n*)). The corresponding best-response dispersal probability *d*_BR_(*n*) is the complement of the natal-case joining probability obtained from the same logistic mapping, *d*_BR_(*n*) = 1 − *P*_join_(*Δ*_natal_(*n*)), where *Δ*_natal_ is evaluated with the outsider relatedness *r*_out_ set to the natal-offspring value $\frac{1\;+\;(n\;-\;1)\;r(n)}{n}$. Natal and floater joining are therefore treated as two cases of a single decision rule that differ only in the relatedness of the focal outsider to the existing insiders. Finally, we then update the candidate strategies towards the best response with a damping factor *η* ∈ (0, 1]: *j*^(t+1)^(*n*) = *j*^(t)^(*n*) + *η* [*j*_BR_(*n*) − *j*^(t)^(*n*)], and analogously for *d*^(t+1)^(*n*). We use *η* = 0.1 throughout. A complete step-by-step specification of this algorithm, together with a justification of the damping step, is given as algorithm S1 in the supplementary material.

## 3. Calculating demographic equilibria and inclusive-fitness pay-offs

Population dynamics in our model can be expressed in terms of three sets of differential equations: (i) the first set describes the rates of change in the frequencies *f(n)* of groups of different sizes and in the number of floaters per territory, *f_d_*, (ii) the second set tracks changes in the reproductive values *v(n)* of individuals in groups of different sizes and of floaters, *v_F_*, and (iii) the third set tracks the mean relatedness *r(n)* among individuals in groups of varying size. Setting the rates of change of the group size frequencies to 0, we solve numerically for the equilibrium frequency distribution, and then use a similar approach to derive reproductive values and relatedness coefficients. The derivation of each set of equations is summarized in the supplementary material. To determine, for instance, the rates of change in the frequencies of groups of a given size, we consider all of the classes of events that lead to the creation of such a group (i.e. birth of a non-dispersing offspring or immigration of floaters into a smaller group, death in a larger group) or loss of such a group (i.e. birth of a non-dispersing offspring or immigration of floaters or death in a group of the focal size), and the rates at which they occur, as tabulated in table S1 in the supplementary material.

The inclusive-fitness gain to a focal individual of joining a group of size *n* is given by

$$\Delta I_{out}\left(n\right)\;=\;\left[\left(1\;+\;r_{out}n\right)v\left(n\;+\;1\right)\right]\;-\;\left[v_{F}\;+\;r_{out}nv\left(n\right)\right],$$

where *r*_out_, the mean relatedness of the focal outsider to the insiders, is equal to 0 when the focal is a floater joining a foreign group, but is given by

$$r_{out}\;=\;\left(1\;+\;\left(n\;-\;1\right)r\left(n\right)\right)/n,$$

when the focal outsider is a juvenile joining its natal group. Similarly, the mean inclusive fitness gained by the insiders is given by

$$\Delta I_{in}\;=\;\left[\left(1\;+\;\left(n\;-\;1\right)r\left(n\right)\;+\;r_{out}\right)v\left(n\;+\;1\right)\right]\;-\;\left[\left(1\;+\;\left(n\;-\;1\right)r\left(n\right)\right)v\left(n\right)\;+\;r_{out}v_{F}\right].$$

All three expressions have simple interpretations. Each square bracket collects an individual's inclusive fitness in one of two states (joining versus not joining). For the outsider (ΔI_out_), the first bracket gives the focal individual's own reproductive value *v*(*n* + 1), earned by living in a group of size *n* + 1 after joining, plus *r*_out_ × *n* copies of *v*(*n* + 1) captured through relatedness to each of the *n* new co-residents. The second bracket gives the alternative: as a floater, the focal contributes *v*_F_ directly, and *r*_out_ × *n* copies of *v*(*n*) through the *n* insiders that remain in their original size-*n* group. The natal-case expression for *r*_out_ reflects that one of the *n* current insiders is the focal's parent (relatedness 1 in our asexual model), while the remaining *n* − 1 are co-residents of the parent at average relatedness *r*(*n*) to the focal, so the mean is [1 + (*n* − 1) *r*(*n*)] / *n*. The insider expression (ΔI*_in_*) is the per-insider analogue: each insider's own value changes from *v*(*n*) to *v*(*n* + 1) on accepting the outsider, and the inclusive-fitness bookkeeping over the *n* − 1 other insiders (at relatedness *r*(*n*) to the focal) and the new outsider (at relatedness *r*_out_ to the focal) yields the displayed expression.

## 4. Results

Our model shows that the degree of membership control (i.e. insider versus outsider control) produces qualitatively different equilibrium group-size distributions: under insider control, groups concentrate near the most productive size, whereas under outsider control they spread towards larger sizes (figures 2 and 3). The patterns of group size distribution, group stability and mean relatedness within groups of different sizes can be better understood by investigating the reproductive values of existing group members and floaters, which, in turn, will influence the dispersal and joining decisions of individuals. In general, an individual's reproductive value is higher in smaller groups and decreases as group size increases (figure 4). This is because small groups are expected to experience positive net growth (figure 5), so individuals in small groups are more likely to transition towards medium-sized groups where *per capita* productivity is higher. By contrast, large groups are expected to shrink because mortality generates frequent losses (figure 5c,g). This net-growth pattern arises because, when groups are small, the combined rates of natal recruitment and floater immigration exceed the mortality-driven loss rate (figure 5a–h). Greater insider control over group membership lowers the reproductive value of floaters (supplementary material, figure S1) because it will be harder for floaters to force individuals to let them join a group if joining lowers the fitness of insiders. As a result, the mean group size is predicted to be smaller, and the group size will be closer to the most productive size if insiders have greater control over group membership.

**Figure 3.**
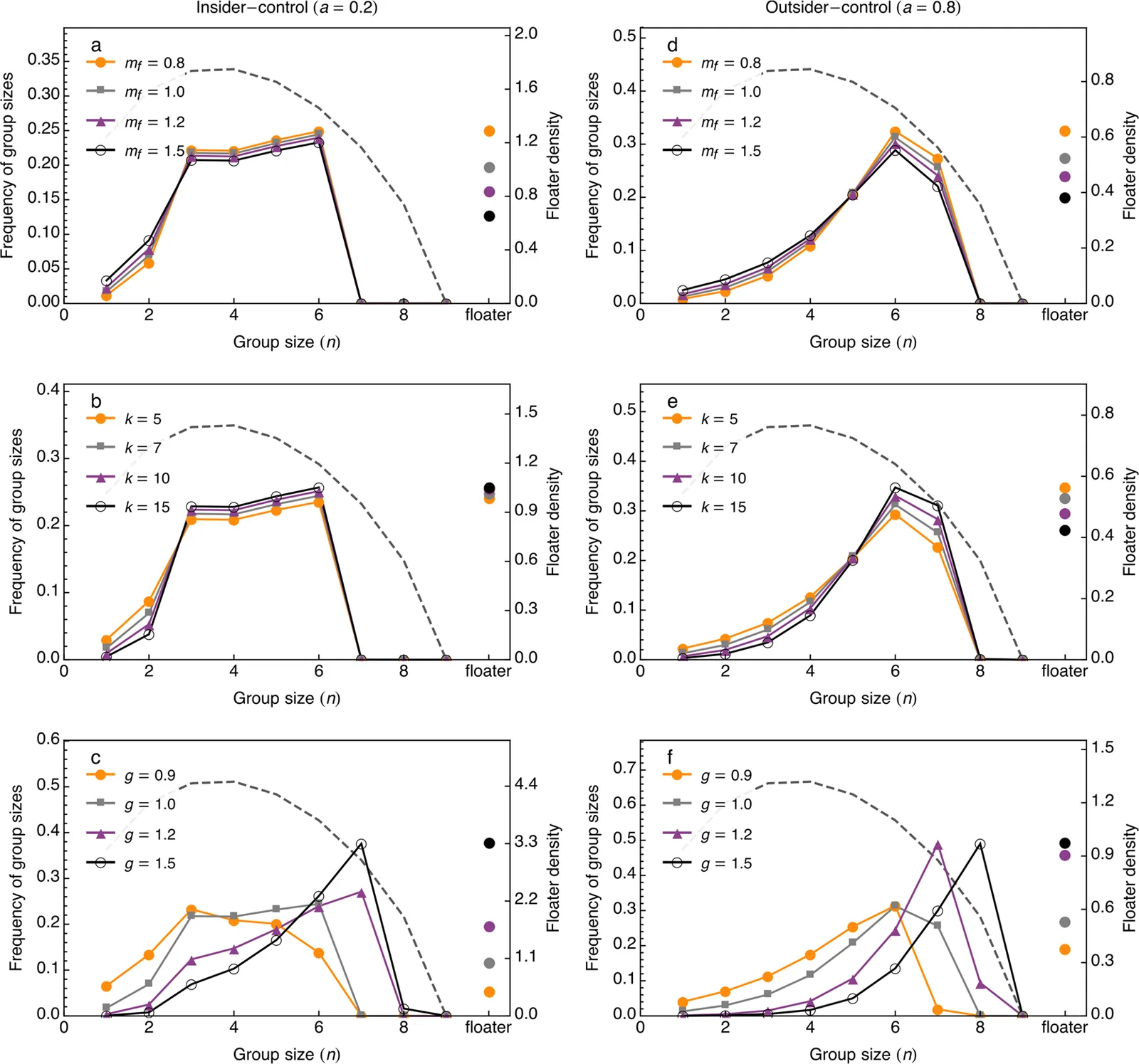
Equilibrium frequency distribution of group sizes and floater density under different demographic conditions. Curves show the equilibrium frequency *f*(*n*) of groups of size *n* (left y-axis). Symbols plotted at the x-axis position labelled ‘floater’ show the equilibrium floater density *f_d_* (mean number of floaters per territory; right y-axis). Left column: insider-control (*a*  =  0.2). Right column: outsider-control (*a*  =  0.8). Rows vary (a,d) floater mortality hazard *m_f_*, (b,e) floater encounter rate *k* and (c,f) fecundity multiplier *g*. Note that the floater density axis scale differs across rows (e.g. panels c and f). This figure uses the same baseline *p*(*n*) function as figure 2. The grey dashed curve in each panel shows the baseline *per capita* productivity *p*(*n*)/*n*, rescaled separately in each panel to 85% of that panel's left-axis maximum (refer to figure 2 for absolute values); the shape of the curve is identical across panels because *p*(*n*)/*n* is independent of *m_f_*, *k* and *g* (*g* rescales *p*(*n*) multiplicatively without shifting the peak location).

**Figure 4.**
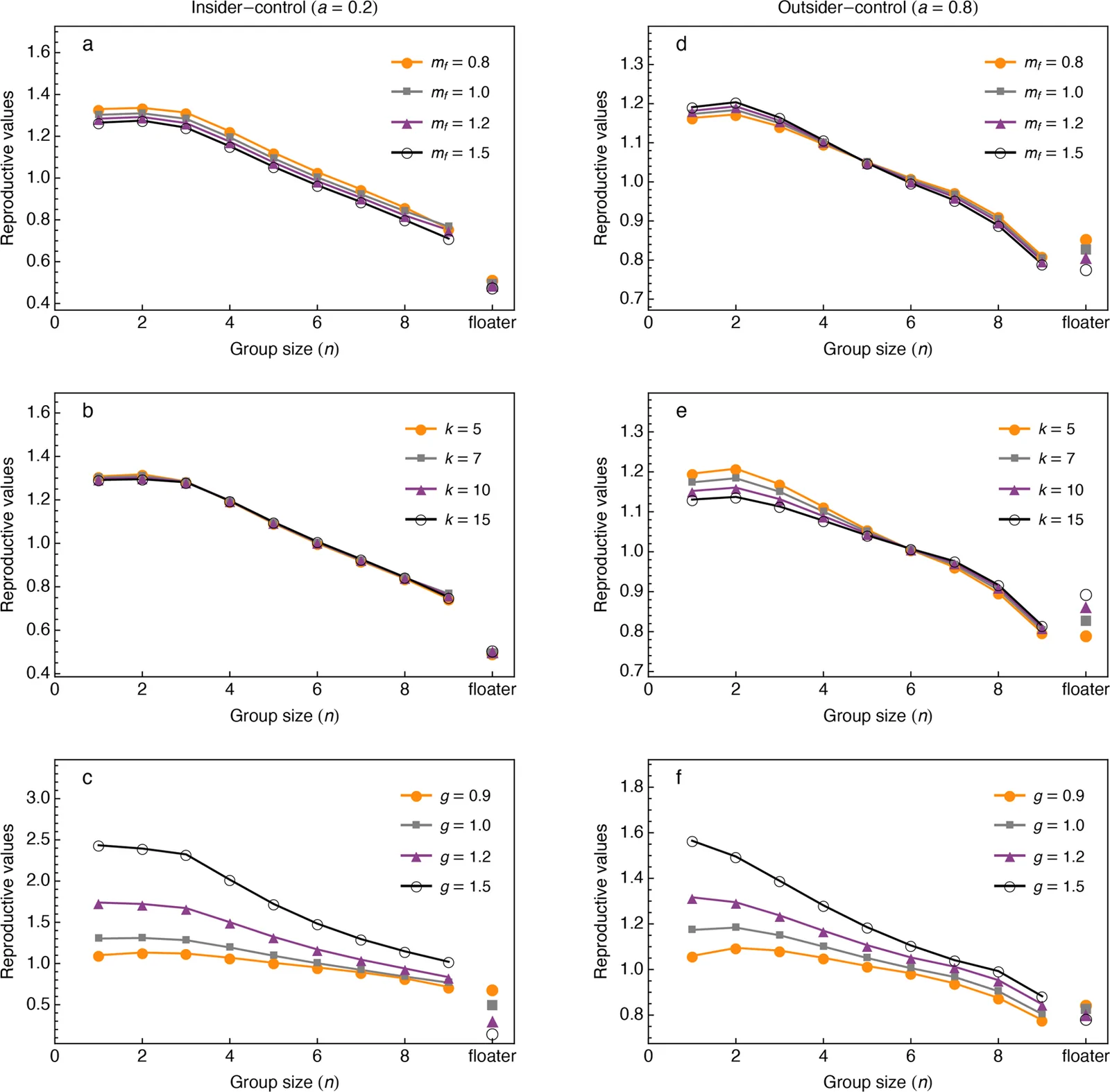
Equilibrium reproductive values as functions of group size under different demographic conditions. Lines show the reproductive value of a resident in a group of size *n*, *v*(*n*). Symbols plotted at the x-axis position labelled ‘floater’ show the reproductive value of a floater, *v_F_*. Left column: insider-control (*a*  =  0.2). Right column: outsider-control (*a*  =  0.8). Rows vary (a,d) floater mortality hazard *m_f_*, (b,e) floater encounter rate *k* and (c,f) fecundity multiplier *g*. Reproductive values are normalized following section 1.3 in the supplementary material, so that values are comparable across scenarios.

**Figure 5.**
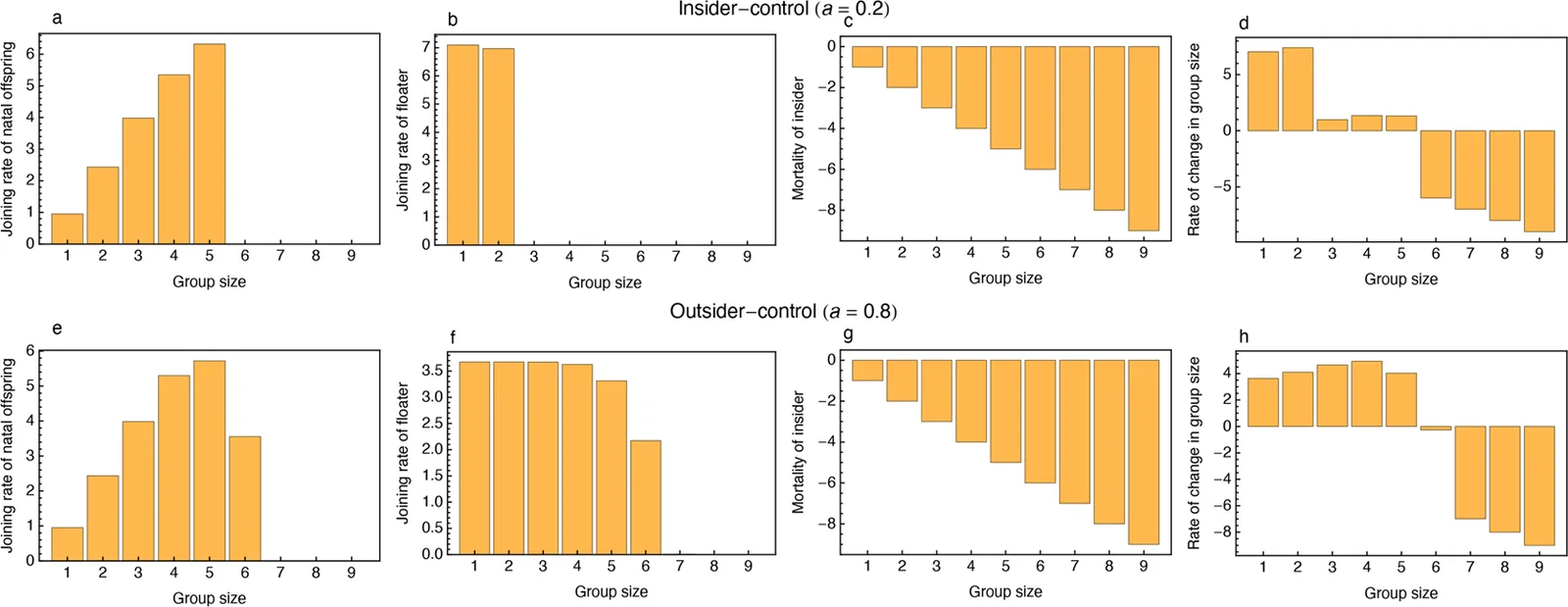
The offspring joining rate, floater joining rate, mortality contribution and net rate of group-size change as functions of group size *n* in the (a–d) insider-control (*a*  =  0.2) and (e–h) outsider-control (*a*  =  0.8) scenarios. All quantities plotted are event rates per unit time (time^–1^), not probabilities. (a, e) Natal-offspring recruitment rate: Λ_natal_ (*n*) =  *p*(*n*) [1 − *d*(*n*)]. (b, f) Floater immigration rate: Λ_floater_ (*n*) = *f_d_*  *k* *j*(*n*). (c, g) Mortality contribution (negative): Λ_death_ (*n*) =   − *nm* (each death event reduces group size by one); plotting this contribution as a negative quantity is a sign convention that allows the net rate in (d, h) to be read as the sum of the three preceding panels. (d, h) Net expected rate of group-size change: Λ_Δ*n*_ (*n*) = Λ_natal_ (*n*) + Λ_floater_(*n*) + Λ_death_(*n*). Because these are rates, they are not bounded by 1; for example, a joining rate of 4 means an expected four recruitment/joining events per unit time (with the time unit defined by setting *m*  =  1).

The stability and distribution of group sizes in the population are determined by three main forces: (i) the number of offspring produced and remaining in the natal group, (ii) the immigration rate of floaters, and (iii) the mortality rate of group members (figure 5). Recall that we consider offspring born in the natal territory as outsiders because their joining still requires acceptance by current group members. We found that there are only minor differences in the number of offspring remaining in the natal group between the insider- and outsider-control scenarios (figure 5a,e). Instead, the differential immigration rate of floaters is the key mechanism generating the differences in the distributions of group sizes between the insider- and outsider-control models. Floaters are only allowed to join groups when group sizes are much smaller than the most productive size in the insider-control model (figure 5b), whereas immigration rates of floaters are predicted to be relatively constant across different group sizes in the outsider-control model (figure 5f). Given that we assume constant *per capita* mortality in groups of various sizes, larger groups will have more death events simply because they contain more individuals (figure 5c,g). Our model therefore predicts that small groups tend to grow, medium-sized groups close to the most productive size are the most stable, and large groups tend to shrink (figure 5d,h). As a consequence, a hump-shaped distribution of group sizes is predicted under our model's assumptions, consistent with patterns commonly observed in nature [20,21,48,49].

Fecundity has a substantial influence on the distribution of group sizes: lower fecundity leads to a better fit between the distributions of group productivity and group size in the insider-control model (figure 3c), whereas higher fecundity results in more large groups (figure 3c,f). This pattern is caused by the fact that higher fecundity leads to higher floater density, which in turn causes the less-productive small groups to quickly become filled up by floaters and increase in size, as well as push the medium-size groups to become larger than the most productive size (figure 3c). For a similar reason, the lower mortality rate of floaters (figure 3a,d) and a higher rate of floaters encountering a group (figure 3b,e) also shift the group size distribution towards larger sizes.

Finally, our models also predict that when insiders control group membership, the mean genetic relatedness among group members will be higher in larger groups when fecundity is low (figure 6a–c; supplementary material, figure S1). Similar to the process influencing the group size distribution, two main effects jointly cause this group size-relatedness pattern: (i) because group productivity is lower in smaller groups, relatively fewer offspring are produced, and thus fewer offspring remain in small groups (figure 5a), and (ii) more floaters are allowed to join when groups are small and less productive (figure 5b). By contrast, when outsiders control group membership, mean relatedness will be low, and group size will have a relatively small but negative effect on average relatedness (figure 6d–f). This is because floaters can join groups of all sizes, but because there are fewer small groups in the population, floaters will join small groups at a lower rate than large groups (figure 5e,f).

**Figure 6.**
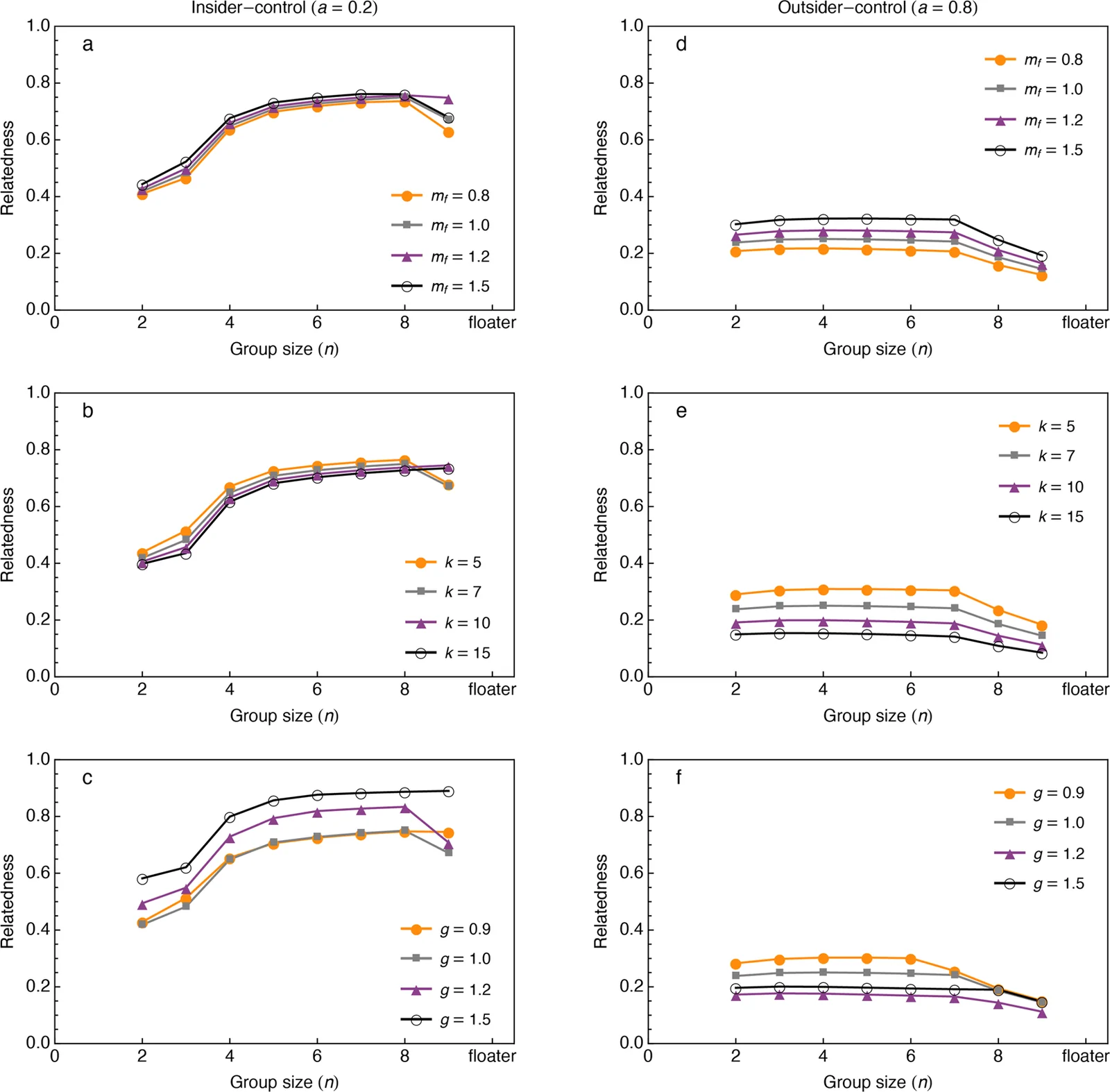
Equilibrium mean within-group relatedness as a function of group size. Curves show *r*(*n*), the mean genetic relatedness among members of groups of size *n*, computed from the equilibrium demographic process (supplementary material, section 1.2). Left column: insider-control (*a*  =  0.2). Right column: outsider-control (*a*  =  0.8). Rows vary (a,d) floater mortality hazard *m_f_*, (b,e) floater encounter rate *k* and (c,f) fecundity multiplier *g*. Curves are shown for all group sizes *n* = 2 to *n*_max_. Note that under insider control, the largest sizes are almost unoccupied at equilibrium (*f*(8) and *f*(9) are below 10^−11^ at the baseline), so the relatedness values plotted there refer to group sizes that essentially do not occur.

In addition, lower floater mortality (figure 6a,d) and higher floater encounter rates of a breeding group (figure 6b,e) will generate lower mean genetic relatedness among group members. This occurs primarily because these conditions increase the influx of unrelated individuals into breeding groups, since floaters from different natal groups are unlikely to be related to existing group members (insiders). Consequently, as floater survival improves and encounter rates increase, the proportion of genetically unrelated individuals within groups rises, diluting overall within-group relatedness. In other words, non-kin groups are more likely to form when floater mortality is low, and floater density is high. Fecundity has the opposite effect in the insider- and outsider-control models. Mean relatedness among group members is predicted to be higher in more productive populations or species when insiders control group membership, whereas the opposite pattern is predicted when outsiders control membership (figure 6c,f). This contrast is caused by two complementary mechanisms. First, differential immigration rates of floaters between the insider- and outsider-control scenarios in the high-fecundity cases allow more floaters to join groups in the outsider-control scenario. Second, because floaters are unrelated to the groups that they join ( *r*_out_ =  0 for foreign-group joining), increasing fecundity changes the balance between natal recruitment and floater immigration. Under insider control, floater admission is largely restricted to small, low-productivity groups (figure 5b), so increases in *g* disproportionately raise the contribution of natal recruitment to group growth, yielding higher within-group relatedness in medium and large groups. Under outsider control, higher *g* also elevates floater density and hence immigration into groups of many sizes (figure 5f), increasing the contribution of unrelated immigrants and thereby reducing relatedness.

## 5. Discussion

We propose that the variation in kinship within social groups can be best understood by viewing the two extremes of most offspring dispersing or staying as two ends of a group-formation continuum. Our model brings together seemingly disparate group properties, such as group productivity, group size, group stability and kin structure and their social and ecological determinants, within a general framework. Key predictions from our model include that (i) in the insider-control model, larger groups will have higher average genetic relatedness if fecundity of the small groups is low and thus kin are not available to join. This counterintuitive pattern arises because floaters are largely excluded from groups at or above the *per capita* productivity optimum, so larger groups grow primarily through natal recruitment, whereas small groups are diluted by both reduced natal output and elevated floater admission. Kin-based admission therefore becomes the binding criterion only when groups reach their optimal size, where the inclusive-fitness cost of accepting unrelated outsiders is sufficient to drive their rejection. Consequently, unrelated individuals are more likely to be allowed to join when the group size is smaller than the optimal size so that groups can reach higher productivity, though relatives will still be allowed to join even when the group size is larger than the optimal size. However, in the outsider-control model, the opposite pattern is expected to occur. In addition, (ii) groups will be most stable when their sizes are close to the most productive size. Further, (iii) group size will converge to slightly larger sizes than the most productive size in the insider-control model, whereas group size will converge to the largest size that is still more productive than for the solitary individuals in the outsider-control model. Finally, (iv) non-kin groups (or mixed-kin groups with lower average relatedness) will be more likely to form if fecundity is low in the insider-control model, whereas high fecundity predicts lower relatedness in the outsider-control model. We discuss the implications of these four predictions in the following sections, noting that they are evaluated at self-consistent demographic equilibria where inclusive-fitness effects reflect long-run state transitions through reproductive values. These predictions are derived under our specific modelling assumptions, most notably asexual reproduction with strategically chosen dispersal and joining decisions, and may not hold in systems with strong behavioural constraints such as obligate natal recruitment or strongly sex-biased philopatry; we discuss this further in the section below.

### 5.1. Variation of within-group relatedness and the evolution of non-kin groups

Perhaps the most puzzling cases of kin structure are those species in which social groups contain many non-kin members (i.e. mixed-kin groups [42]), given that every living organism must have relatives and the genetic interests of relatives are more aligned than those of unrelated individuals. Our model shows that the crucial reasons favouring the formation of non-kin or mixed-kin groups in the insider-control model are factors promoting offspring dispersal, such as high floater survival rates and high rates of breeding vacancy encountered by floaters. Under this scenario of insider control, dispersing offspring have relatively high fitness, which results in the formation of non-kin groups. Alternatively, even when most offspring stay on their natal territory, factors that prevent groups from reaching the most productive size, such as low group fecundity and high mortality of group members, can also favour non-kin groups in the insider-control model. Essentially, relatives alone cannot form groups that are productive enough, and thus, we predict that mean genetic relatedness among groups will be more variable in this scenario.

Preliminary support for this prediction comes from two long-term studies of obligate cooperatively breeding species, the white-winged chough [50,51] and the superb starling [42]. In normal years, groups of white-winged choughs are formed primarily through offspring delaying dispersal, and it has been well established that cooperation between breeders and helpers is essential for them to breed successfully owing to the difficulty of provisioning young. However, a severe drought significantly reduced within-group relatedness in white-winged choughs because high adult mortality caused groups to re-form via the amalgamation of surviving adults from different groups [50]. In addition, white-winged choughs living in suburban areas also had higher adult mortality owing to human activities, and, as predicted by our model, the average genetic relatedness of the social groups of white-winged choughs living in these harsher, suburban environments was also lower than that of the groups in natural forests [51]. In superb starlings, which live in highly unpredictable environments characterized by frequent droughts, groups cannot produce enough offspring to maintain size. Only through the acceptance of unrelated outsiders can groups maintain stability in the face of environmental fluctuation [42]. Thus, even in species characterized by offspring delayed dispersal and high relatedness, environmentally driven demographic stochasticity can still cause considerable variation in the genetic structure of social groups.

Previous studies have often treated within-group relatedness *r* as an explanatory variable measured independently of the ecological conditions that determine *b* and *c* (the benefit and cost terms of Hamilton's rule). Our model shows that those same conditions can also alter *r* through demographic processes and behavioural membership decisions. This emergent view of relatedness complements recent reaffirmations of the centrality of relatedness in social evolution [52], by showing how relatedness coefficients themselves arise from the demographic and behavioural processes that structure group formation.

We are not the first to derive relatedness from demographic processes; earlier work (e.g. [8]) has done so within structured-population frameworks. Our specific contribution is to embed this derivation within an explicit model of insider-outsider conflict over group membership, where dispersal and joining strategies, group size and relatedness co-evolve at demographic equilibrium. For example, our model shows that the degree of membership control and fecundity have interacting effects on within-group relatedness. Empirical studies have mostly focused on species in which offspring tend to stay in the natal territory when fecundity is high, which in turn causes greater within-group relatedness [30,53,54]. That is, if insiders have high control over group membership, social groups will comprise closely related kin, but if insiders' control of group membership is not strong, groups will mainly be composed of non-relatives because the immigration pressure from floaters will also be higher [19].

Our model also predicts that, in species living in variable and unpredictable environments, the interests of insiders and outsiders are more likely to align. When mortality is high, and the persistence of groups is uncertain, insiders benefit from admitting unrelated outsiders to prevent group failure, even when they retain full control over membership [42]. Indeed, recent empirical work by Shah & Rubenstein [55] provides compelling evidence for this prediction in a cooperatively breeding bird species, where they demonstrate that environmental quality influences patterns of dispersal, creating a gradient of group structures within the same population. Specifically, superb starlings (*Lamprotornis superbus*) exhibit directional dispersal from groups in lower-quality habitat to groups in higher-quality habitat, leading to smaller kin-based groups in resource-poor areas and larger mixed-kin groups in resource-rich areas. In addition, the finding in the banner-tailed kangaroo rat (*Dipodomys spectabilis*) that there was a negative association between the fine-scale genetic structure (i.e. kin clustering in the free-entry case) and population density (representing fecundity) among years provides good preliminary support for our model prediction [56]. With the rapid development of molecular tools, we believe that the time is ripe for more detailed studies examining the ecological and demographic causes of variations in kin structure.

### 5.2. Group size and relatedness

By explicitly incorporating the feedback effects among dispersal, joining strategies and population dynamics, our model predicts that when insiders control group membership, mean relatedness among group members will generally increase as group size increases, to the point that the *per capita* productivity of the group is close to, but higher than, the productivity of solitary individuals. This is because not only will fewer offspring be produced and stay in natal groups, but more floaters will also be allowed to join small groups. By contrast, relatedness will be generally low and decrease as group size increases when outsiders control group membership because floaters can join groups of all sizes.

To the best of our knowledge, these predictions have not been explicitly tested in any system. Some empirical evidence has shown that relatedness decreases as group size increases in group-living species [57]. However, whether groups are loosely formed (e.g. in free entry foraging groups) or groups are relatively stable (e.g. in cooperatively breeding groups) is unclear [57]. A study of brood-rearing coalitions in cooperatively breeding common eiders provides preliminary support for these predictions [58]. In this system, older, more experienced females, which are likely to exert stronger control over coalition composition, formed smaller coalitions composed of more closely related individuals, whereas younger or less dominant females formed larger coalitions with lower within-group relatedness. Mapped onto our framework, this contrast corresponds to a gradient in the control parameter a: stronger insider control (lower effective a) yields the insider-control prediction of smaller, more-related groups concentrated near the productivity optimum, whereas weaker insider control (higher effective a) yields larger, less-related groups consistent with the outsider-control regime. This demographic and group size variation occurs within a single population and reflects differences in dominance and experience at an individual level, rather than differences between species. This is precisely the kind of within-species social variation that our model is designed to explain. Nevertheless, more empirical studies are clearly needed to understand the relationship between group size and genetic structure within social groups.

### 5.3. Optimal and equilibrium group size and group stability

Our model also provides a general framework for studying the distribution of group sizes and of group stability. In addition to testing whether observed group sizes are larger than the most productive size, it will also be fruitful to study the detailed mechanisms influencing group formation, the replacement of group members and the magnitude and direction of group size changes. These questions are crucial for understanding the conflict of interests over group membership among group members and its resolution, yet they have rarely been integrated within a unified inclusive-fitness framework that links demography, kin structure and dispersal/joining decisions, despite extensive long-term empirical documentation of these dynamics in cooperatively breeding species like meerkats [59,60], acorn woodpeckers [61,62] and cichlid fishes [63,64]. For example, eviction, which usually involves dominant individuals evicting subordinates through overtly aggressive behaviour, clearly suggests that there is conflict over group composition because one party will rarely leave voluntarily, yet the other party can often benefit from reducing the group's size [65–67]. Our model provides a synthetic framework for investigating how group productivity, genetic relatedness and population demography jointly influence the resolution of insider-outsider conflict and impact group size in species in which eviction occurs.

Our analysis focuses on self-consistent demographic equilibria under stationary, group-size–dependent joining rules. Inclusive-fitness effects are evaluated using reproductive values computed under the stationary demographic process induced by these rules; consequently, the pay-off of accepting (or rejecting) an outsider incorporates long-run consequences through expected future transitions in group size and relatedness, rather than only immediate changes in productivity. Nevertheless, we do not model explicit within-lifetime forward planning, nor non-stationary strategies that depend on richer state variables (e.g. the full kin composition of a group beyond mean relatedness). Exploring such dynamic strategic feedback would be a valuable extension of the present framework.

A further limitation concerns the biological scope of our framework. We have assumed asexual reproduction throughout, which simplifies the bookkeeping of relatedness and reproductive value but ignores the often substantial differences between male and female dispersal regimes in sexual species. In many vertebrates that breed cooperatively, philopatry is strongly sex-biased, with typically one sex remaining in the natal group while the other disperses. Group composition reflects the combined effects of sex-specific philopatry and lineage-level recruitment rules. The present framework can be extended to these systems by treating *d*(*n*) as sex- or lineage-specific (with separate dispersal and joining strategies for each sex) and propagating the resulting relatedness asymmetries through the reproductive-value calculation; the *r*(*n*) recursion in section 1.2 in the supplementary material generalizes naturally to a matrix of pairwise relatedness coefficients indexed by class. Similarly, obligate natal recruitment (as in some clonal or eusocial species) corresponds to fixing *d*(*n*) ≡ 0 for the natal-philopatric sex and leaving the floater-joining machinery to operate only on the dispersing sex. These extensions preserve the core mathematical structure but require additional bookkeeping that we defer to future work.

In conclusion, although kin groups are commonly found in nature [54] and expected by inclusive-fitness theory, recent studies have revealed that there is greater variation in kin structure than previously thought. For example, nearly half of cooperatively breeding bird species [30] and mammal species [68] form social groups that contain non-kin helpers or co-breeders. Nevertheless, the causes of the variation in kin structure within and among different populations or species have long been overlooked, presumably owing to the lack of a theoretical framework for exploring this question [58,69,70]. Our model provides a theoretical framework for exploring group size, group stability and kinship composition using inclusive-fitness theory. It shows how kin structure can be an emergent outcome of demographic processes and membership decisions. By treating relatedness as an emergent property of these processes rather than as a fixed input, the model generates testable predictions for how kin structure, group size and social stability should covary across demographic and ecological gradients. We hope that the theoretical framework presented here will stimulate more theoretical and empirical attention to these important but largely neglected issues of kinship dynamics of groups and, in turn, lead to a more complete understanding of social evolution.

## Supplementary Material



## Data Availability

All code is provided as supplementary material [71].
