# Supplementary material for: The evolution of group size and kin structure in complex societies
Source: R Soc Open Sci. 2026 Oct 28;13(10):260096. doi: 10.1098/rsos.260096 (PMC13714408; doi:10.1098/rsos.260096)
Supplement: Supplementary file 1 [file rsos.260096.zip › rsos.260096/rsos260096_si_001.docx]

**Electronic Supplementary Material (ESM)**

**The evolution of group size and kin structure in complex societies**

Sheng-Feng Shen, Ming Liu and Dustin R. Rubenstein

**ESM Text**

We model an asexual population as a continuous-time, Moran-style event-based process, in which the productivity, dispersal rate of offspring, and joining probability of floaters are functions of group size, $n$. Unlike the classical Moran model, population size is not fixed. Instead, the size of each social group can vary from 0 to $n_{max}$, and we list all the corresponding rate of events of each group size in Table S1. We note that at maximum group size (i.e., $n_{max}$= 9), the offspring always disperse and floaters are never accepted. These rates are used in calculating stable frequencies of group size, the relatedness among group members, and reproductive values in the following sections.

**Table S1. List of parameters and rates for each type of group.**

| Group size | Frequency | Mortality | Productivity | Dispersal (offspring) | Accepting a floater | Offspring stays in natal patch |
| --- | --- | --- | --- | --- | --- | --- |
| 0 | $f_{0}$ | N/A | N/A | N/A | $f_{0}f_{d}j_{0}k$ | N/A |
| 1 | $f_{1}$ | $m$ | $p_{1}$ | $d_{1}$ | $f_{1}f_{d}j_{1}k$ | $(1-d_{1})f_{1}p_{1}$ |
| 2 | $f_{2}$ | $2m$ | $p_{2}$ | $d_{2}$ | $f_{2}f_{d}j_{2}k$ | $(1-d_{2})f_{2}p_{2}$ |
| 3 | $f_{3}$ | $3m$ | $p_{3}$ | $d_{3}$ | $f_{3}f_{d}j_{3}k$ | $(1-d_{3})f_{3}p_{3}$ |
| 4 | $f_{4}$ | $4m$ | $p_{4}$ | $d_{4}$ | $f_{4}f_{d}j_{4}k$ | $(1-d_{4})f_{4}p_{4}$ |
| 5 | $f_{5}$ | $5m$ | $p_{5}$ | $d_{5}$ | $f_{5}f_{d}j_{5}k$ | $(1-d_{5})f_{5}p_{5}$ |
| 6 | $f_{6}$ | $6m$ | $p_{6}$ | $d_{6}$ | $f_{6}f_{d}j_{6}k$ | $(1-d_{6})f_{6}p_{6}$ |
| 7 | $f_{7}$ | $7m$ | $p_{7}$ | $d_{7}$ | $f_{7}f_{d}j_{7}k$ | $(1-d_{7})f_{7}p_{7}$ |
| 8 | $f_{8}$ | $8m$ | $p_{8}$ | $d_{8}$ | $f_{8}f_{d}j_{8}k$ | $(1-d_{8})f_{8}p_{8}$ |
| 9 | $f_{9}$ | $9m$ | $p_{9}$ | $d_{9}=1$ | $f_{9}f_{d}j_{9}k=0$ | $\left( 1-d_{9} \right)f_{9}p_{9}=0$ |
| floater | $f_{d}$ | $m$_f_ | N/A | N/A | N/A | N/A |

In each column of Table S1, we can see the general forms of rates of events: $p_{n}$ for productivity, $d_{n}$ for dispersal rate, $f_{n}f_{d}j_{n}k$ for the rate at which floaters are accepted (i.e., *j_n_* is the per-encounter probability that a floater is accepted; $f_{n}f_{d}j_{n}k$ is the corresponding population-level event rate for size-n groups), and $(1-d_{n})f_{n}p_{n}$ for an offspring to stay in its natal group. We note that mortality is $nm$ instead of m because we assume that mortality is the same for all residents, so the total rate at which a group of size $n$ loses one individual is $nm$ (because $m$ is a per-capita hazard rate).

## 1.1 Parameters, events, and change in group frequencies

We begin by finding the equilibrium frequencies $f_{n}$ for each group size $n$ (ranging from 0 to $n_{\max}$) in this continuous-time, Moran-style framework. At equilibrium, the rate at which a focal group size $n$ increases must balance the rate at which it decreases. Specifically, a group of size $n$ can increase in frequency if a group of size $n-1$ accepts a floater, if a group of size $n-1$ reproduces and retains its offspring, or if a group of size $n+1$ loses one individual through mortality and becomes $n$. Conversely, a group of size $n$ can decrease in frequency if it accepts a floater (thus becoming $n+1$), if it reproduces and retains the offspring (also becoming $n+1$), or if it loses one individual to mortality (becoming $n-1$). Denoting the mortality hazard rate as $m$ per individual, and the group-level birth rate as $p_{n}$ in a group of size $n$, and the probability of dispersal for new offspring as $d_{n}$, we write the net change of $f_{n}$ as:

$$\Delta f_{n}=f_{n-1}f_{d}j_{n-1}k+\left( n+1 \right)f_{n+1}m_{n+1}+\left( 1-d_{n-1} \right)f_{n-1}p_{n-1}-f_{n}f_{d}j_{n}k-nf_{n}m_{n}-\left( 1-d_{n} \right)f_{n}p_{n},$$

where the terms in the first line increase $f_{n}$, and those in the second line decrease $f_{n}$. Throughout this paper, we set m*_n_* ≡ m for all n (constant per-capita mortality across group sizes); the subscript notation in the equation is retained for generality. The quantity $f_{d}$ indicates the density of floaters (the average number of floaters per territory), $j_{n}$ is the probability that a floater is accepted by a group of size $n$, and $k$ is the rate at which floaters encounter groups. In addition to solving $\Delta f_{n}=0$ for all $n$, it is necessary to impose that $\sum_{i=0}^{n_{\max}} f_{i}=1$ and that there is no net change in floater density, $\Delta f_{d}=0$. The latter condition can be written as:

$$\Delta f_{d}=-\sum_{i=0}^{n_{\max}} f_{i}f_{d}j_{i}k-f_{d}m_{f}+\sum_{i=1}^{n_{\max}} d_{i}f_{i}p_{i}=0,$$

reflecting losses of floaters by joining groups and by mortality, and gains of floaters from dispersing offspring.

This expression is used with the boundary conventions *f*_−1_ = *f*_nmax+1_ = 0, *p*_0_ = 0, and the imposed constraints *d*_nmax_ = 1, *j*_nmax_ = 0; equivalently, in size-*n*_max_ groups all offspring disperse and floaters are never admitted. Because the *Δf_n_* equations are linearly dependent (their sum is zero by conservation of territory number), we solve *Δf_n_* = 0 for *n* = 0, …, *n*_max_ by replacing one redundant equation with the normalization ∑_n_ *f_n_* = 1, together with *Δf_d_* = 0.

## 1.2 Calculating relatedness at each group size

After solving for the stable frequencies $f_{n}$, the next step is to calculate the equilibrium relatedness $r_{n}$ among members of each group size $n$. This requires tracking how events that produce a new group of size $n$ (that is, from $n-1$ or $n+1$) contribute to the average relatedness among its members. In particular, only those events that create a group of size $n$ affect $r_{n}$. These events are the production of a non-dispersing offspring in a group of size $n-1$, the acceptance of a floater by a group of size $n-1$, and the loss of one individual from a group of size $n+1$. The equilibrium relatedness $r_{n}$ can be expressed by assigning the appropriate relatedness to each event and weighting by the rates at which those events occur:

$$r_{n}=\frac{\left( 1-d_{n-1} \right)f_{n-1}p_{n-1}\left( \frac{2}{n}\left[ \frac{1}{n-1}+\frac{n-2}{n-1}r_{n-1} \right]+\frac{n-2}{n}r_{n-1} \right)+f_{n-1}f_{d}j_{n-1}k\left( \frac{n-2}{n}r_{n-1} \right)+\left( n+1 \right)f_{n+1}m_{n+1}r_{n+1}}{\left( 1-d_{n-1} \right)f_{n-1}p_{n-1}+f_{n-1}f_{d}j_{n-1}k+\left( n+1 \right)f_{n+1}m_{n+1}}.$$

The numerator weights each event's rate by the average pairwise relatedness in the post-event group of size *n*. A group of size *n* contains C(*n*, 2) = *n*(*n* − 1)/2 distinct pairs, so *r*(*n*) is a weighted average of pairwise identity-by-descent (IBD) probabilities across these pairs. The three events that create a group of size *n* — birth into a size (*n* − 1) group, acceptance of a floater into a size (*n* − 1) group, and loss of one individual from a size (*n* + 1) group — are decomposed as follows.

***Birth event*** (at rate (1 − *d*_n−1_) *f*_n−1_ *p*_n−1_). The new offspring is asexually produced by one of the *n* − 1 original members. Among the *n*(*n* − 1)/2 pairs of the post-event group, *n* − 1 contain the new individual and the remaining (*n* − 1)(*n* − 2)/2 do not. The fraction of pairs that contains the new individual is therefore 2/*n* — this is the meaning of the leading 2/*n* factor in the formula: a pair-counting probability, not an IBD probability. Within those pairs, exactly one is the parent–offspring pair (IBD = 1) and the remaining *n* − 2 link the new individual to a non-parent co-resident of the parent, with relatedness *r*(*n* − 1) (because the new individual is an asexual clone of its parent, its relatedness to anyone equals the parent's). The expected IBD across pairs that contain the new individual is therefore $\frac{1 + (n - 2) r(n - 1)}{n - 1}$. The complementary fraction (*n* − 2)/*n* of pairs do not contain the new individual, and their expected IBD is the prior average *r*(*n* − 1).

***Floater-acceptance event*** (at rate *f*_n−1_ *f*_d_ *j*_n−1_ *k*). The same pair-counting decomposition applies, but the joining floater is unrelated to the existing members. The 2/*n* fraction of pairs that involve the new individual therefore contribute IBD = 0; the complementary (*n* − 2)/*n* fraction contribute *r*(*n* − 1). The net contribution to *r*(*n*) from this event is (*n* − 2)/*n* · *r*(*n* − 1).

***Mortality event*** (at rate (*n* + 1) *f*_n+1_ *m*_n+1_). One individual is removed at random from a group of size *n* + 1; the average pairwise relatedness among the *n* survivors equals the average pairwise relatedness of the original size-(*n* + 1) group, *r*(*n* + 1), because uniformly random removal preserves the average over pairs. The denominator of the recursion is the total event rate (1 − *d*_n−1_) *f*_n−1_ *p*_n−1_ + *f*_n−1_ *f_d_* *j*_n−1_ *k* + (*n* + 1) *f*_n+1_ *m*_n+1_, weighting the three contributions. Relatedness is undefined for a solitary individual (*n* = 1), which contains no within-group pair, and for an empty group (*n* = 0); the recursion above does not use r(1), and every summary reported in the figures begins at n = 2.

## 1.3 Calculating reproductive values

Once the stable frequencies and relatedness values have been determined, the reproductive values $v_{n}$ for groups of size $n$ can be calculated. In a continuous-time Moran framework, the expected reproductive value of an individual in a group of size $n$ is governed by the demographic events that can occur within that group, each of which changes the focal individual’s reproductive value. Four main events are considered: mortality (which takes the group from $n$ to $n-1$), acceptance of a floater ($n$ to $n+1$), birth of a non-dispersing offspring ($n$ to $n+1$), and birth of a dispersing offspring (leaving the group size unchanged). Denoting these events’ rates as before, the condition for equilibrium is:

$$nf_{n}m\left( -v_{n}+\frac{n-1}{n}v_{n-1} \right)+f_{n}f_{d}j_{n}k\left( -v_{n}+v_{n+1} \right)+\left( 1-d_{n} \right)f_{n}p_{n}\left( -v_{n}+\frac{n+1}{n}v_{n+1} \right)+d_{n}f_{n}p_{n}\frac{v_{F}}{n}=0.$$

The first term corresponds to mortality. An individual has a $1/n$ chance of being the one that dies (in which case its reproductive value goes to 0) and a $\left( n-1 \right)/n$ chance of remaining in a group of size $n-1$. The second term reflects how accepting a floater changes the group size to $n+1$, which shifts the individual’s reproductive value from $v_{n}$ to $v_{n+1}$. The third term represents the birth of a non-dispersing offspring, which not only changes the group from $n$ to $n+1,$ but also gives the parent an additional contribution if it is the one producing the new individual, captured by the factor $\left( n+1 \right)/n v_{n+1}$. The fourth term involves the birth of a dispersing offspring, which does not alter the focal group size and thus does not subtract the original $-v_{n}$. Because the parent is determined with probability $1/n$, it adds $v_{F}/n$ in expectation, where $v_{F}$ is the reproductive value of a floater. Two extra conditions are used to close the system. First, the total reproductive value (weighted by the group frequencies) is set to one:

$$\frac{\sum_{i=1}^{n_{\max}} if_{i}v_{i}+f_{d}v_{F}}{\sum_{i=1}^{n_{\max}} if_{i}+f_{d}}=1.$$

Second, there is no net change in the overall reproductive value of floaters:

$$\sum_{i=0}^{n_{\max}-1} f_{i}j_{i}k\left( -v_{F}+v_{i+1} \right)-m_{f}v_{F}=0.$$

Solving these equations simultaneously yields the equilibrium reproductive values $v_{n}$ for each group size and $v_{F}$ for floaters.

**Table S2.** Parameter values used to generate the figures. Values in parentheses are the alternatives used in the corresponding parameter sweeps (Figures 3, 4 and 6). All other parameters were held at the values in this table.

| **Symbol** | **Value used** | **Meaning** |
| --- | --- | --- |
| *n*_max_ | 9 | Maximum group size |
| *m* | 1 | Resident mortality hazard per capita, constant across group sizes; sets the time unit |
| *p*_0_(*n*) | 0.2 *n*^1.5^ (9 − *n*)^0.75^ | Baseline group productivity for *n* = 1, …, *n*_max_ |
| *g* | 1 (0.9, 1.0, 1.2, 1.5) | Fecundity multiplier, *p*(*n*) = *g* *p*_0_(*n*) |
| *m*_f_ | 1 (0.8, 1.0, 1.2, 1.5) | Floater mortality hazard per capita |
| *k* | 7 (5, 7, 10, 15) | Floater–group encounter rate |
| *a* | 0.2 / 0.8 | Extent of outsider control, used for the insider-control and outsider-control panels; Figure S1 sweeps *a* = 0, 0.01, …, 1 |
| *ε* | 0.025 | Decision uncertainty in *P*_join_; the equivalent tanh form used in the implementation takes err = 2ε = 0.05 |
| *η* | 0.1 | Damping factor of the best-response update |
| *d*(*n*) at *t* = 0 | 0.5 for *n* < *n*_max_; *d*(*n*_max_) = 1 | Initial dispersal probabilities |
| *j*(*n*) at *t* = 0 | 1 for *n* < *n*_max_; *j*(*n*_max_) = 0 | Initial joining probabilities |
| *T* | 1000 | Number of best-response iterations |
| Software | Wolfram Mathematica 12.0 | Implementation; see the archived code (Appendix S1 and Appendix S2) |

**ESM Figure**

**Figure S1**. Consequences of varying the degree of outsider control, $a\in[0,1]$, while holding all other parameters at the baseline values listed in Table S2. (a) Reproductive value of floaters, $v_{F}$, as a function of $a$. (b) Mean within-group relatedness (pair-weighted average across multi-member groups, n ≥ 2), $\bar{r}=\frac{\sum_{n=2}^{n_{\max}} n\left( n-1 \right) f_{n}r_{n}}{\sum_{n=2}^{n_{\max}} n\left( n-1 \right) f_{n}}$. (c) Pearson correlation between group size and within-group relatedness across multi-member group sizes, weighted by the equilibrium group-size frequencies *f*(*n*) so that each group size contributes in proportion to how often it occurs at equilibrium (computed across $n\geq2$, with the horizontal dashed line indicating zero correlation). Relatedness is undefined for a solitary individual, so *n* = 1 is excluded from both summaries. Curves are evaluated at *a* = 0, 0.01, …, 1. The step-like features arise because the equilibrium floater-joining probabilities for successive group sizes (*j*_2_, *j*_3_, *j*_4_, …) switch from near 0 to near 1 at successive thresholds in *a*, shifting the group-size distribution upwards in discrete increments. The size–relatedness correlation remains positive across most of the control gradient and changes sign only under near-complete outsider control, between *a* = 0.78 and *a* = 0.79.

**Methods S1: Numerical Procedure and Reproducibility**

# **Algorithm S1. Iterative numerical procedure for self-consistent equilibrium**

## **Overview**

This algorithm describes the iterative numerical workflow used to obtain a self-consistent demographic equilibrium and strategy equilibrium. The workflow corresponds to the update function (e.g., upd) iterated by a wrapper function (e.g., run) using repeated application (e.g., Nest) in Mathematica.

## **Inputs**

- Maximum group size: $n_{\max}=9$.
- Resident mortality vector: $\mathbf{m}=\{m_{1},\ldots,m_{n_{\max}}\}$ (per-capita hazard rates, time^−1^).
- Group productivity vector: $\mathbf{p}=\{p_{1},\ldots,p_{n_{\max}}\}$ (group-level birth rates, time^−1^).
- Floater mortality: $m_{f}$ (per-capita hazard rate, time^−1^).
- Encounter rate: $k$ (time^−1^).
- Control parameter: $a\in\left[ 0,1 \right]$.
- Decision uncertainty: *ε* = 0.025 in *P*_join_(Δ) = 1/(1 + exp(−Δ/*ε*)); the equivalent tanh form used in the implementation takes err = 2*ε* = 0.05.
- Initial strategies:
  - Dispersal strategy $\mathbf{d}^{\left( 0 \right)}=\{d_{1}^{\left( 0 \right)},\ldots,d_{n_{\max}-1}^{\left( 0 \right)},1\}$ with boundary $d_{n_{\max}}\equiv1$.
  - Floater acceptance strategy $\mathbf{j}^{\left( 0 \right)}=\{j_{0}^{\left( 0 \right)},\ldots,j_{n_{\max}-1}^{\left( 0 \right)},0\}$ with boundary $j_{n_{\max}}\equiv0$.
- Step size (damping) $\eta\in(0,1]$ (required; we use η = 0.1).
- Iteration limit $T$, and/or convergence tolerances (recommended below).

## **Outputs**

- Final strategies $\mathbf{d}^{*},\mathbf{j}^{*}$.
- Equilibrium demographic quantities: group-size distribution $\mathbf{f}=\{f_{0},\ldots,f_{n_{\max}}\}$ and floater density $f_{d}$.
- Equilibrium relatedness values $\mathbf{r}=\{r_{2},\ldots,r_{n_{\max}}\}$.
- Equilibrium reproductive values for residents $\mathbf{v}=\{v_{1},\ldots,v_{n_{\max}}\}$ and floaters $v_{F}$.

## **Procedure (pseudocode)**

1. **Initialize:** set $\mathbf{d}\leftarrow\mathbf{d}^{\left( 0 \right)}$ and $\mathbf{j}\leftarrow\mathbf{j}^{\left( 0 \right)}$.
2. **Repeat for** $t=0,1,\ldots,T-1$ **(or until convergence):**
   a. **Demographic equilibrium.** Given $\mathbf{d}$ and $\mathbf{j}$, solve the demographic balance equations for the stationary group-size distribution $\mathbf{f}$ and floater density $f_{d}$ using a numerical root-finder (e.g., FindRoot). The demographic process is continuous-time Moran-style: events occur one-at-a-time; a death event removes exactly one individual; therefore group size changes by at most one per event ($n\to n\pm1$).
   b. **Relatedness equilibrium.** Using $\left( \mathbf{f},f_{d} \right)$, solve the relatedness recursion to obtain $\mathbf{r}=\{r_{2},\ldots,r_{n_{\max}}\}$ (e.g., via a coupled system solved by FindRoot).
   c. **Reproductive values.** Using $\left( \mathbf{f},f_{d} \right)$, solve the reproductive-value system to obtain resident reproductive values $\mathbf{v}=\{v_{1},\ldots,v_{n_{\max}}\}$ and floater reproductive value $v_{F}$ (e.g., via a coupled system solved by FindRoot).
   d. **Compute best responses.** Using *f*, *fd*, *r*, *v*, *vF*, and the control parameter *a*, evaluate for each group size *n* the inclusive-fitness change of admitting an outsider, in the *collective* form

Δ(n) = a · ΔIout(n) + (1 − a) · n · ΔIin(n),

where ΔIout and ΔIin are computed from the manuscript expressions using the boundary value *r*_out_, which is set to $\frac{1 + (n - 1) r(n)}{n}$ for a natal joiner (yielding *Δ*_natal_(*n*)) and to 0 for a floater (yielding Δ*floater*(*n*)). The *n* multiplier on ΔIin reflects the fact that each of the *n* insiders experiences the change, and matches the description in the main text. Apply the logistic mapping (taking the complement for the natal case, since *d* denotes dispersal rather than joining)

*d*_BR_(n) = 1 − *P*_join_(*Δ*_natal_(n)), *n* = 1,…,*n*_max_ − 1,

*j*_BR_(n) = *P*_join_(Δ*floater*(n)), *n* = 0,…,*n*_max_ − 1,

with the empty-territory convention *Δ*_floater_(0) = *v_1_* − *v_F_* and *j*_BR_(0) = *P*_join_(*v_1_* − *v_F_*), reflecting that empty territories have no insiders to exert membership control. For *n* ≥ 1, *Δ*_floater_(*n*) follows the weighted form *a* *ΔI*_out_(*n*) + (1 − *a*) *n* *ΔI*_in_(*n*) with *r*_out_ = 0.

where, equivalently in our implementation, *P*_join_(Δ) = 1 / (1 + exp(−Δ / ε)) = (1 + tanh(Δ / (2ε))) / 2, matching the main-text definition; the tanh form is shown only as an equivalent expression.

e. **Update strategies (damped best response).** Update each strategy by a damped step toward its best response:

*d*_n_^(t+1)^ ← *d*_n_^(t)^ + η · [*d*_BR_(n) − *d*_n_^(t)^], *n* = 1,…,*n*_max_ − 1,

*j*_n_^(t+1)^ ← *j*_n_^(t)^ + η · [*j*_BR_(n) − *j*_n_^(t)^], *n* = 0,…,*n*_max_ − 1,

Because d(t) and dBR(n) both lie in [0, 1] and η ∈ (0, 1], the updated values remain in [0, 1] automatically, so no additional clipping is applied. Enforce the boundary constraints d*_nmax_* ≡ 1 and j*_nmax_* ≡ 0 at every iteration. Damping (rather than replacing strategies wholesale with the current best response) is required because the demographic equilibrium and the strategies are mutually coupled: undamped updates (η = 1) can produce oscillations in which, for example, fully admitting floaters at one iteration causes groups to become too large at the new equilibrium, eliciting a best response at the next iteration that admits none, which in turn causes groups to become too small, and so on. With η sufficiently small (we use η = 0.1, equivalent to a 90 % / 10 % mixture of the old strategy and the current best response at each iteration) the iteration approaches a fixed point at which the strategies are simultaneously best responses to the demographic process they themselves generate; the extent to which this is achieved is reported below.

2. **Convergence.** The implementation used for the figures iterates for a fixed number of steps T rather than stopping early; convergence was verified afterwards against both criteria:
   - **Strategy change is small:** $\max_{n}\left| d_{n}^{\left( t+1 \right)}-d_{n}^{\left( t \right)} \right|<\tau_{d}$ and $\max_{n}\left| j_{n}^{\left( t+1 \right)}-j_{n}^{\left( t \right)} \right|<\tau_{j}$ (e.g., $\tau_{d}=\tau_{j}={10}^{-6}$),
   - **Demographic residual is small:** the maximum absolute residual of the demographic equilibrium equations is less than $\tau_{\mathrm{eq}}$ (e.g., $\tau_{\mathrm{eq}}={10}^{-8}$).

Achieved for every parameter combination reported in the figures: the maximum absolute residual was 6.7×10⁻¹⁵ for the demographic equilibrium equations, 1.3×10⁻⁵ for the relatedness recursion, and 2.2×10⁻¹⁵ for the reproductive-value system, at *T* = 1000. The strategy iteration reached a fixed point (maximum one-step change below *τ* = 10⁻⁶) for all strategy components carrying appreciable demographic weight. A small residual oscillation persists in *d*(*n*_max_ − 1) and *j*(*n*_max_ − 1), the probabilities governing entry to the maximum group size; because the equilibrium frequency of that size is of order 10⁻¹³ or smaller under all reported parameter combinations, this oscillation does not affect any reported quantity. We verified this directly: at the parameter values with the largest residual oscillation, every quantity plotted in Figure S1 changed by less than 3×10⁻⁶ between *T* = 1000 and *T* = 10000.

3. **Return.** With the converged strategies *d** and *j**, re-solve the demographic equilibrium (yielding the group-size distribution *f* = {*f*_0_, …, *f_n_*_max_} and floater density *f_d_*), the relatedness recursion (yielding *r* = {*r*_2_, …, *r_n_*_max_}), and the reproductive-value system (yielding *v* = {*v*_1_, …, *v_n_*_max_} and floater reproductive value *v_F_*) one final time using *d** and *j**. Output {*d**, *j**, *f*, *f_d_*, *r*, *v*, *v_F_*}.
